# Combining a Universal Telomerase Based Cancer Vaccine With Ipilimumab in Patients With Metastatic Melanoma - Five-Year Follow Up of a Phase I/IIa Trial

**DOI:** 10.3389/fimmu.2021.663865

**Published:** 2021-05-11

**Authors:** Elin Aamdal, Else Marit Inderberg, Espen Basmo Ellingsen, Wenche Rasch, Paal Fredrik Brunsvig, Steinar Aamdal, Karen-Marie Heintz, Daniel Vodák, Sigve Nakken, Eivind Hovig, Marta Nyakas, Tormod Kyrre Guren, Gustav Gaudernack

**Affiliations:** ^1^ Department of Oncology, Oslo University Hospital, Oslo, Norway; ^2^ Institute of Clinical Medicine, Faculty of Medicine, University of Oslo, Oslo, Norway; ^3^ Department of Oncology, Akershus University Hospital, Lørenskog, Norway; ^4^ Department of Cellular Therapy, Oslo University Hospital, Oslo, Norway; ^5^ Department of Tumor Biology, Institute for Cancer Research, Oslo University Hospital, Oslo, Norway; ^6^ Ultimovacs Allmennaksjeselskap (ASA), Oslo, Norway; ^7^ Centre for Cancer Cell Reprogramming, Institute of Clinical Medicine, Faculty of Medicine, University of Oslo, Oslo, Norway; ^8^ Center for Bioinformatics, Department of Informatics, University of Oslo, Oslo, Norway

**Keywords:** telomerase (hTERT), peptide vaccine immunotherapy, ipilimumab, melanoma, phase I/IIa studies

## Abstract

**Background:**

Ipilimumab improves survival for patients with metastatic malignant melanoma. Combining a therapeutic cancer vaccine with ipilimumab may increase efficacy by providing enhanced anti-tumor immune responses. UV1 consists of three synthetic long peptides from human telomerase reverse transcriptase (hTERT). These peptides comprise epitopes recognized by T cells from cancer patients experiencing long-term survival following treatment with a first-generation hTERT vaccine, and generate long-lasting immune responses in cancer patients when used as monotherapy. The objective of this trial was to investigate the safety and efficacy of combining UV1 with ipilimumab in metastatic melanoma.

**Patients and Methods:**

In this phase I/IIa, single center trial [NCT02275416], patients with metastatic melanoma received repeated UV1 vaccinations, with GM-CSF as an adjuvant, in combination with ipilimumab. Patients were evaluated for safety, efficacy and immune response. Immune responses against vaccine peptides were monitored in peripheral blood by measuring antigen-specific proliferation and IFN-γ production.

**Results:**

Twelve patients were recruited. Adverse events were mainly diarrhea, injection site reaction, pruritus, rash, nausea and fatigue. Ten patients showed a Th1 immune response to UV1 peptides, occurring early and after few vaccinations. Three patients obtained a partial response and one patient a complete response. Overall survival was 50% at 5 years.

**Conclusion:**

Treatment was well tolerated. The rapid expansion of UV1-specific Th1 cells in the majority of patients indicates synergy between UV1 vaccine and CTLA-4 blockade. This may have translated into clinical benefit, encouraging the combination of UV1 vaccination with standard of care treatment regimes containing ipilimumab/CTLA-4 blocking antibodies.

## Introduction

Ipilimumab is a monoclonal antibody that inhibits CTLA-4, permitting rapid expansion of T cells primed by antigen presenting cells (1) and was the first treatment to show a survival benefit in metastatic melanoma (2). The effect depends on a pre-existing immune response recognizing the tumor (1). Clinical benefit from ipilimumab is associated with high tumor mutational burden (3) (TMB) and a high number of predicted neoantigens (4, 5). Hence, priming anti-tumor immune responses by therapeutic cancer vaccines with tumor-related antigens before or during treatment may improve outcomes with checkpoint inhibitors (6).

However, a landmark study found no clinical benefit of combining ipilimumab with a cancer vaccine targeting gp100 in melanoma patients (2), discouraging further clinical trials of such combinations. As the gp100 vaccine was composed of a short synthetic peptide designed to elicit CD8 T cell responses, and ipilimumab primarily affects the expansion of CD4 Th cell responses (7), we decided to test the effect of ipilimumab in the context of a peptide vaccine comprising long peptides with CD4 epitopes. Furthermore, the gp100 vaccine regimen differed from the UV1 vaccination in the use of adjuvant. In the landmark study, Incomplete Freund’s Adjuvant (IFA) was used, whereby an antigen depot is created at the site of vaccination, resulting in trapping of vaccine-specific T cells at the injection site as shown in an animal model (8). In the current trial, we used GM-CSF as an adjuvant and compensated for the lack of depot effect by more frequent vaccinations.

UV1 is a therapeutic cancer vaccine, consisting of three synthetically produced long peptides, primarily inducing CD4+ T helper type 1 (Th1) cells, targeting human telomerase reverse transcriptase (hTERT). Telomerase is expressed in cancer cells at every stage of tumor evolution, from the cancer stem cell to circulating tumor cells and implicated in human cell immortalization and cancer cell pathogenesis (9), proposing a unique cancer antigen as a basis for immunotherapy (10, 11). Based on data from long-term cancer survivors treated with an unrelated first-generation hTERT vaccine, three novel long hTERT peptides were selected for the next generation vaccine, UV1. Immune responses to these peptides were associated with clinical benefit and strong Th1 responses (i.e. secretion of interferon-γ, tumor necrosis factor-α, and IL-2) (12, 13). The UV1 peptides are predicted to contain multiple HLA epitopes (12, 13), providing a potential universal vaccine independent of prior selection based on HLA-typing. UV1 has been investigated in clinical phase I trials in metastatic prostate cancer (14) and NSCLC (15). The scientific rationale for combining UV1 with ipilimumab is based on the potential of the vaccine to generate *de novo* immune responses in cancer patients, thus, broadening the anti-tumor repertoire in patients. Secondly, eliminating the negative effect CTLA-4 has on vaccine-induced T cells, ipilimumab may unleash the true clinical potential of a vaccine (16). Furthermore, ipilimumab reduces immunosuppression in the tumor microenvironment by blocking CTLA-4 on T regulatory cells (1). This phase I/IIa trial explores the potential synergistic effect of CTLA-4 blockade and hTERT vaccination, allowing for unchecked expansion of hTERT-specific T cell clones, in HLA-unselected patients with metastatic malignant melanoma.

## Patients and Methods

### Patients

Patients aged ≥ 18 years with a histologically confirmed diagnosis of unresectable stage III/IV cutaneous malignant melanoma, Eastern Cooperative Oncology Group (ECOG) performance status of 0 or 1, and adequate renal, hepatic and hematological function were eligible for inclusion. Any previous treatment was accepted. Exclusion criteria included active brain metastases, history of autoimmune disease, splenic surgery or irradiation, allogeneic stem cell transplantation, known hypersensitivity to investigational products, positive serologic tests for HIV, syphilis, hepatitis B, or hepatitis C, uncontrolled infectious disease, pregnancy and breastfeeding. All patients have provided written informed consent. Recruitment was planned for 20 participants. The trial was conducted in accordance with the ethical principles of the Declaration of Helsinki and the International Conference on Harmonization of Good Clinical Practice and approved by an independent ethics committee and the appropriate national and institutional review boards.

### Study Design

This was an open-label, single-armed, single-center phase I/IIa clinical trial [NCT02275416]. The primary objective was to investigate the safety of combining UV1 with ipilimumab in patients with unresectable metastatic melanoma. Secondary objectives were to assess immune responses to UV1 peptides, overall response rate (ORR), and overall survival (OS) and progression free survival (PFS).

### Treatment

UV1 (Ultimovacs ASA, Oslo, Norway) consists of three peptides, one 30-mer (p719-20) and two 15-mers (p725 and p728) in equimolar amounts. UV1 was produced as a sterile aqueous solution of drug substances, stored (lyophilized) at minus 20°C (+/- 5°C) and reconstituted in water for injection and stored at 2-8°C for use within 6 hours.

UV1 vaccines of 300 µg doses were administered as intradermal abdominal injections before and between treatments of ipilimumab, and thereafter every fourth week up to 28 weeks, and at week 36 and 48 (**Supplementary Figure S1**) unless clinical deterioration or unacceptable toxicity was encountered. Adjuvant GM-CSF (sargramostim 75 µg) in the form of preservative-free powder (lyophilized Leukine, Sanofi Aventis, Bridgewater, NJ, US) was reconstituted with water and injected intradermally at the same site 10-15 minutes prior to UV1. Ipilimumab (3 mg/kg) was administered every 3 weeks for a total of 4 doses as labeled. Trial drugs were handled by the hospital pharmacy according to standard procedures.

### Clinical Assessment

Safety was evaluated by physical examination and blood sampling at each treatment visit and 30 days after administration of the last dose of UV1. Adverse events (AEs) were assessed according to the National Cancer Institute Common Terminology Criteria for Adverse Events (CTCAE) version 4.0.

Computed tomography (CT) was conducted at baseline, week 12, 16, and 24 after the first dose of ipilimumab, and then every 3 months until disease progression. Tumor response was evaluated using RECIST v1.1 (17). OS was defined as the time from treatment initiation to death, and PFS as the time from treatment initiation to objective tumor progression or death. Survival was censored on December 1, 2020.

### Immunological Assessment

Peripheral blood mononuclear cells (PBMCs, 50 mL in acid citrate dextrose tubes) were obtained from peripheral blood derived at baseline, 2, 6, and 10 weeks after the first vaccine, and then, every 4 weeks. The UV1-specific proliferative response was determined using two vials of thawed PBMCs per time point as described previously (14). PBMCs were stimulated with UV1 vaccine peptides 725 (hTERT 691-705), 719-20 (hTERT 660-689), and 728 (hTERT 651-665) (Bachem AG, Switzerland) at a concentration 10 µM for each peptide. On day 12, cells were re-stimulated with peptide and tested for proliferation by ^3^H-thymidine incorporation assays as described previously (14). The stimulation index (SI) was calculated using mean counts of wells containing T cells and irradiated antigen presenting cells (APCs) loaded with UV1 peptide divided by mean counts of wells without peptide. An SI ≥ 3 was considered as a positive response. Blood was analyzed for the presence of IgE specific for GM-CSF and the UV1 peptides using an enzyme-linked immunosorbent assay (ELISA)-based in-house method described in **Supplementary Figure S2**. If cell numbers were sufficient, IFN-γ ELISPOT assays were performed with pre-stimulated PBMCs from the cultures set up for proliferation as previously described (14).

HLA genotyping was performed retrospectively for each patient by Prolmmune Ltd Tissue Typing Service, using Tier 1 Typing by PCR-sequence specific oligonucleotides (PCR-SSOP) to resolve major allele groups to 4 digits with some degeneracy (e.g. HLA-A*23:01/03/05/06).

### Estimation of Tumor Mutational Burden (TMB)

Biopsies were harvested at baseline and week 12. 20 mg of the biopsy was disrupted on a TissueLyser LT followed by DNA extraction using AllPrep DNA/RNA/miRNA Universal kit on the Qiacube (Qiagen). Whole-exome sequencing was performed on all available biopsies and analyzed for TMB.

Exome library preparation was conducted with 1 ug DNA as starting material and using the Agilent AllExome v5 kit, according to the vendor´s protocol. Libraries were sequenced paired-end (2x150 bp), generating approximately 90 M PE reads per tumor and 40 M PE reads per normal, using SBS chemistry on a HiSeq4000 system. Variant calling was performed essentially as described previously (18) (**Supplementary Methods**). TMB was considered low at (1-5 mutations/Mb), intermediate at (6-19 mutations/Mb) and high at (≥20 mutations/Mb).

## Results

### Patient Characteristics and Treatment

Patients were recruited between January and October 2015. Inclusion was terminated after twelve of the planned 20 patients were enrolled, as PD-1 inhibitors replaced ipilimumab as standard first-line treatment for metastatic melanoma (November 2015). Patient baseline characteristics are described in **Table 1**. The median age was 57 (44-74) years. All, but one patient, were ECOG 0. Five were female (42%) and seven male (58%). Three patients (25%) were M1a, two M1b (17%), six M1c (50%) and one M1d (8%) according to AJCC 8^th^ Ed. Six patients (50%) had elevated LDH. Three patients (25%) were *BRAF* ^V600E^ mutation positive and nine (75%) mutation negative. Eight patients were treatment naïve and four patients had received one previous line of treatment, including two patients who received vemurafenib and two patients who received dacarbazine. No patients had prior immunotherapy.

**Table 1:** Patient baseline characteristics.

Patient	Age	Sex	ECOG	Stage^a^	Metastatic sites	*BRAF* ^V600E^ genotype	LDH >UNL	TMB	Previous treatment	Disease status at inclusion
N01	47	F	0	M1c	Liver, lungs, bone, lymph nodes, subcutaneous	Negative	Yes	8.6		PD
N02	49	M	1	M1a	Lymph nodes, subcutaneous	Positive	Yes	5.5	Vemurafenib	PD
N03	74	M	0	M1c	Bone, lymph nodes, subcutaneous	Negative	No	87.2		PD
N04	61	M	0	M1c	Lymph nodes, spleen	Positive	Yes	NA	Vemurafenib	PD
N05	44	M	0	M1c	Bone, liver	Negative	No	9.0	Dacarbazine	SD
N06	72	F	0	M1a	Skin	Negative	No	57.2		PD
N07	57	F	0	M1c	GI tract, liver, lung, lymph nodes, soft tissues	Negative	Yes	38.7		PD
N08	65	M	0	M1b	Lung	Negative	No	NA		PD
N09	57	F	0	M1c	Lung, adrenal glands	Negative	Yes	73.8		PD
N11	58	M	0	M1b	Lung, lymph nodes	Negative	No	1.7	Dacarbazine	PD
N13	57	F	0	M1a	Lymph nodes	Negative	Yes	2.1		PD
N14	52	M	0	M1d	CNS, lymph nodes, subcutaneous	Positive	No	NA		PD

^a^Metastatic stage according to American Joint Commission on Cancer 8^th^ Edition ECOG, Eastern Cooperative Oncology Group; M-stage, Metastatic stage; LDH, Lactate dehydrogenase; ULN, Upper limit normal; TMB, Tumor mutational burden; NA, not available; PD, progressive disease; SD, stable disease.

A mean of 5.5 UV1 vaccinations (3-9) and 3.2 (1-4) courses of ipilimumab were administered (**Supplementary Table S1**). Due to a safety concern in a concurrent UV1 trial in metastatic hormone-naive prostate cancer (14), vaccination was temporarily interrupted in October 2015, but did not affect ipilimumab treatment. As an Independent Data Monitoring Committee deemed further treatment safe, vaccination was continued. However, the Norwegian Medicines Agency required a substantial protocol amendment, and to comply, vaccination was again stopped November 2015. At the time of protocol amendment approval (March 2016), no further vaccinations were scheduled.

### Safety

Safety was assessed for all patients. Treatment was generally well tolerated, with most AEs being grade 1-2 (**Table 2**). 95 adverse events were reported, of which 78 were considered related. The most commonly reported treatment-related toxicities were diarrhea, injection site reaction, pruritus, rash, nausea and fatigue. Five patients experienced treatment-related grade 3 toxicity; diarrhea, colitis and rectal hemorrhage, nausea, hypersensitivity, and hypophysitis and dehydration, respectively. No grade 4-5 AEs were reported. Ten serious adverse events (SAEs) were reported in five patients and included hypophysitis, colitis, diarrhea, duodenitis, rectal hemorrhage, dehydration, hypersensitivity, dermatitis, dysarthria, and wound infection. Dysarthria and wound infection were considered unrelated to treatment, whereas the remaining were considered related. Duodenitis, wound infection and dermatitis were classified as grade 2; otherwise, SAEs were classified as grade 3.

**Table 2:** Treatment related adverse events in the safety population.

Treatment Related Adverse Events	Patients (%^a^)				
	Grade			Total	
Gastrointestinal disorders	1	2	3	n	%
Diarrhea	2	3	1	6	50
Colitis		1	1	2	17
Lower GI hemorrhage			1	1	8
Duodenitis		1		1	8
Abdominal pain		1		1	8
Constipation	1			1	8
Nausea		2	1	3	25
Vomiting	1			1	8
**General disorders and administration site conditions**					
Injection site reaction	4	1		5	42
Fatigue	2	1		3	25
Influenza like illness	1	1		2	17
Chills	2			2	17
Edema peripheral	2			2	17
**Skin and subcutaneous tissue disorders**					
Pruritus	4	1		5	42
Rash	2	2		4	33
**Endocrine disorders**					
Hypophysitis	1		1	2	17
**Eye disorders**					
Vision blurred		1		1	8
**Immune system disorders**					
Hypersensitivity			1	1	8
**Metabolism and nutrition disorders**					
Anorexia		1		1	8
Dehydration			1	1	8
**Musculoskeletal and connective tissue disorders**					
Arthralgia		1		1	8
**Nervous system disorders**					
Dizziness		1		1	8

^a^rounded to the nearest number Patients could have more than one adverse event.

One patient experienced a hypersensitivity reaction following the ninth vaccination and completion of four courses of ipilimumab. Symptoms resolved with dexchlorpheniramine 5 mg IV, hydrocortisone 100 mg IV, and salbutamol 2.5 mg inhaled. Further UV1 vaccination in the patient was withdrawn. However, retrospective analyses of blood from this patient revealed increased levels of GM-CSF-specific IgE, but not IgE specific for UV1 peptides (**Supplementary Figure S2**). Ipilimumab was withdrawn due to toxicity in three patients experiencing hypophysitis (one) and colitis (two). One patient skipped the second course of ipilimumab due to dysarthria considered unrelated to treatment. Otherwise, toxicity did not cause treatment interruption in patients.

### Tumor Response

**Figure 1:**
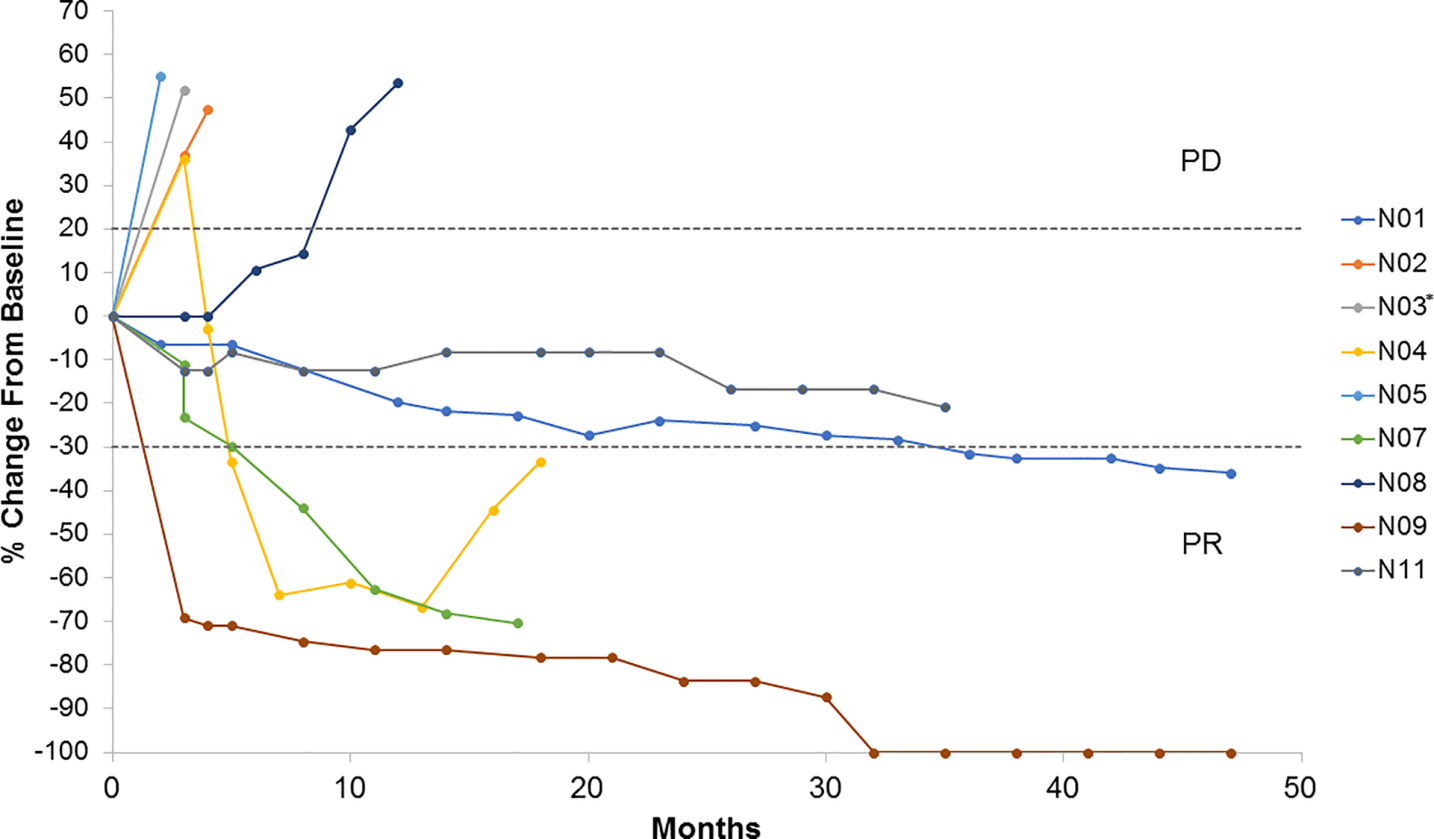
Tumor growth by subject. Spider plot illustrating changes in target lesions from baseline in patients evaluable by RECIST v.1.1 (*N*=9). *Patient N03 was non-evaluable at 12 weeks PR, partial response; PD, progressive disease.

**Figure 2:**
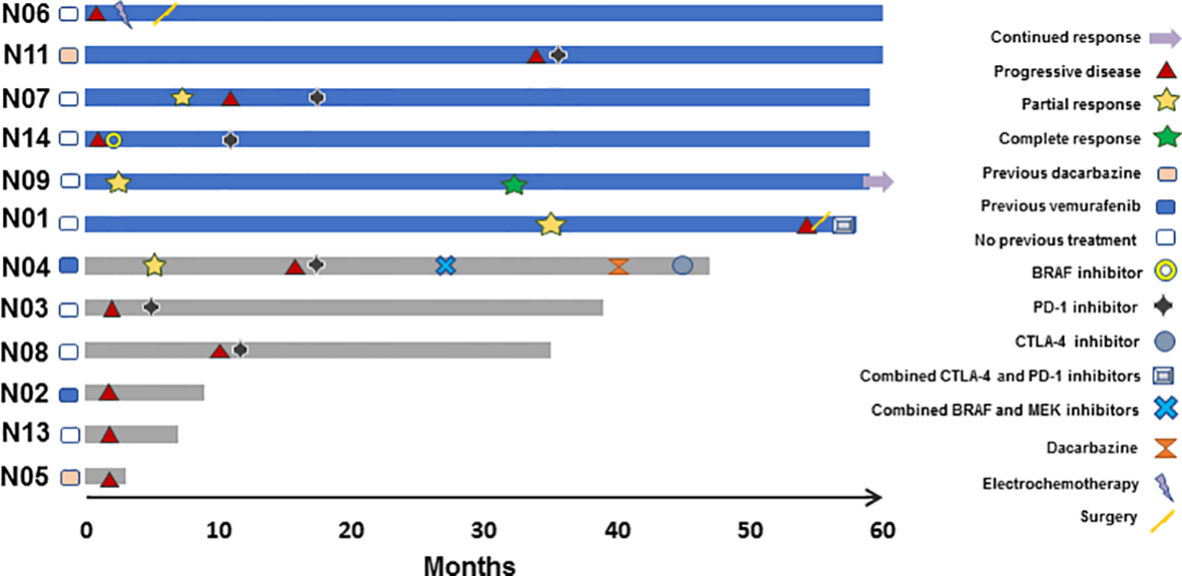
Efficacy assessment by subject censored on December 1 2020. Swimmers plot depicting individual patients as lines, illustrating duration of overall survival in months. Blue and grey colors indicate alive and deceased patients, respectively (*N*=12). Responses and new systemic treatment are indicated by designated symbols. Patient N06 received locoregional chemotherapy and surgery, but no further systemic treatment.

Nine patients were evaluable for tumor response according to RECIST 1.1. As best overall response (BOR), one patient achieved a complete response (CR), three a partial response (PR), two stable disease (SD), and three progressive disease (PD) as illustrated in **Figure 1**. Patients, who were not evaluable according to RECIST 1.1, progressed clinically. Thus, the ORR was 33%. Five patients stopped treatment due to PD. One patient has an ongoing CR as of December 1, 2020 (**Figure 2**). All patients evaluable for tumor response were immune responders.

### Survival

Median follow-up was 61.0 months. Three patients died within one year of study entry. No deaths were attributed to study treatment. Median OS was not reached. OS was 75% at 1 and 2 years, 67% at 3 years and 50% at 5 years. Median PFS was 6.7 months. PFS was 33% at 1 year and 25% at 2 years.

### Immune Response

**Figure 3:**
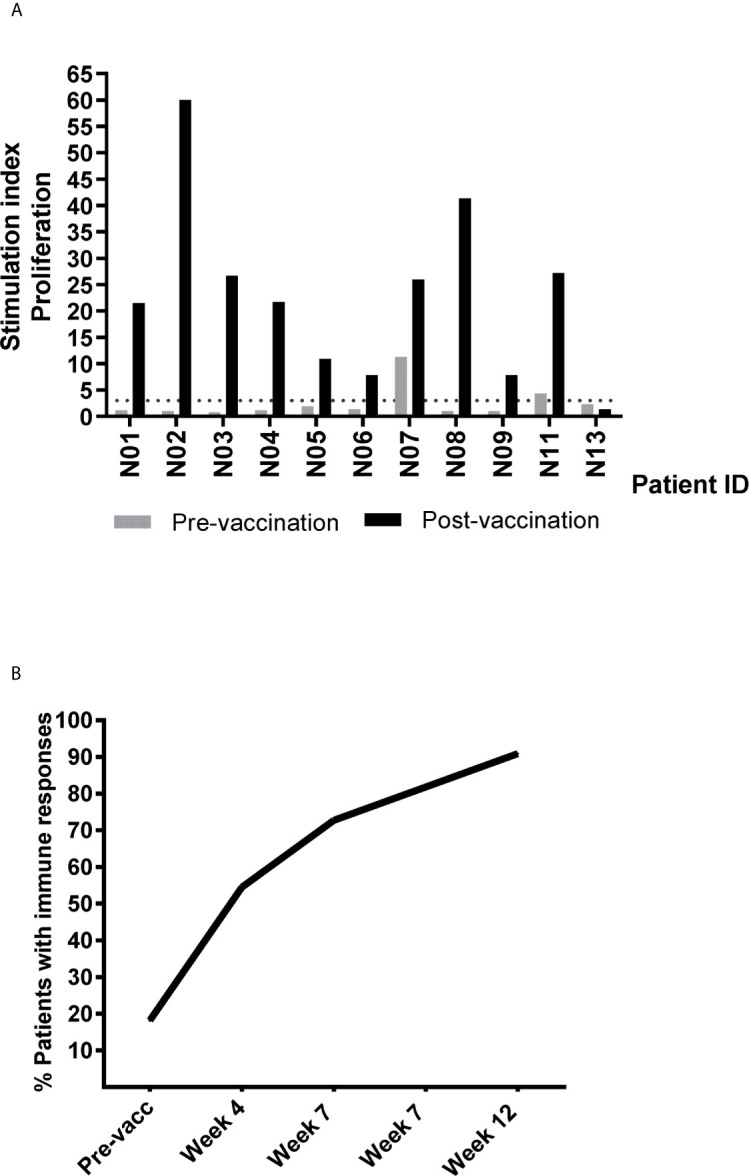
Summary of detected pre- and post-vaccination T cell responses against UV1 peptides. **(A)** T cell proliferation against UV1 peptides in pre- and post-vaccination blood samples from patients evaluable for immune response, depicting the strongest post-vaccination T cell response detected against the hTERT peptide mix for each patient. (*N*=11). Proliferation was measured in response to peptide-loaded PBMC by ^3^H-thymidine incorporation. A stimulation index (SI) of >3 was considered as an immune response. The dotted line indicates SI=3. **(B)** Cumulative percentage of evaluable patients exhibiting immune responses to UV1 peptides. Immune responses at baseline were detected in two patients.

**Figure 4:**
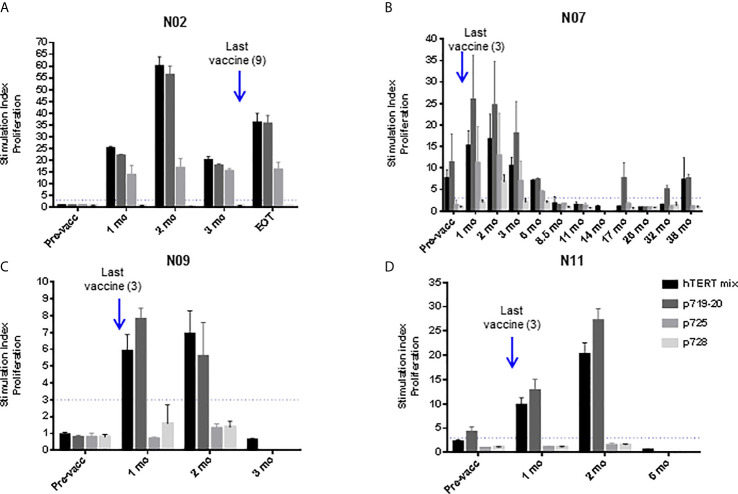
UV1-specific proliferation by T cells at different time points post-vaccination. UV1 peptide-specific proliferation was measured (counts per minute, cpm) at several sampling time points and representative examples are shown for patient N02 **(A)**, patient N07 **(B)**, patient N09 **(C)** and patient N11 **(D)**. Proliferation was measured in response to peptide-loaded PBMC by ^3^H-thymidine incorporation. A 3-fold increase in proliferation compared to non-peptide control (T+ APC) was considered as an immune response. Superantigen SEC-3 stimulation was included as a positive control. Dotted line indicates cut-off for positive response, measurements until last positive time point is shown.

Eleven patients were evaluable for immune response. One patient was judged non-evaluable due to the lack of post-vaccination samples. UV1-specific T cell responses were recorded in ten out of eleven evaluable patients (91%) (**Figure 3A** and **Supplementary Figure S3**). The patient who did not demonstrate an immune response had only one post-vaccination sample at four weeks. All immune responders demonstrated an immune response to the UV1 peptide mix, with a median SI of 23.8 (7.8-60). Two patients exhibited spontaneous, pre-vaccine responses to UV1 peptides and developed a more pronounced and/or broader immune response during treatment, while the remaining developed de-novo responses elicited by vaccination. Six patients exhibited UV1-specific immune responses after four weeks and ten after twelve weeks (**Figure 3B**). When analyzing immune responses to individual peptides, different patterns were seen, exemplified by proliferation responses in four patients (**Figure 4**). In the same patients, cell numbers were also sufficient to perform IFN-γ ELISPOT assays at certain time points, largely demonstrating a correlation between the UV1-specific proliferative response and IFN- γ production (**Supplementary Figure S4**). Unfortunately, there was insufficient patient material to separate CD4 and CD8 T cells for assessment by proliferation and ELISPOT assays. However, T cell cloning of responding cells from one patient (results not shown) and intracellular cytokine staining assessed by flow cytometry in the lung cancer UV1 vaccine study (15) confirmed that the majority of responding T cells were CD4+.

Retrospective analyses showed a wide repertoire of HLA alleles in the study population, and immune responses were seen across different HLA types (**Supplementary Table S2**).

### TMB Estimations

Biopsies were harvested from nine patients at baseline and from four patients at week 12. Baseline TMB is reported in **Table 1**. Median TMB was 9 mutations/Mb. Two patients were considered to have a low TMB, three patients intermediate, and four patients high. There was no obvious correlation between baseline TMB and clinical response (TMB high vs. TMB non-high). Of the four patients with matching biopsies, two patients achieved PR and two PD as BOR. The two responders experienced considerable post-treatment reductions in TMB, of 9 to 1 mutations/Mb and 39 to 11 mutations/Mb, respectively, whereas the two patients who progressed demonstrated no considerable change.

## Discussion

Patient baseline characteristics in this trial indicate that the study population is representative and comparable to other reports on ipilimumab in metastatic melanoma (2, 19, 20). This phase II trial demonstrates that combining UV1 and ipilimumab is safe with mainly low-grade toxicity including diarrhea, nausea, injection site reaction, pruritus, rash, and fatigue. With the exception of injection site reaction, these are well-known side effects of ipilimumab (2, 19, 20). Low-grade gastrointestinal and skin toxicities were slightly more frequently encountered than in ipilimumab monotherapy. Injection site reactions in the current trial were more commonly reported than in UV1 monotherapy (14), maybe reflecting enhanced immunity. Otherwise, we found no increased toxicity combining ipilimumab with UV1.

One patient experienced a hypersensitivity reaction with bronchospasm after the ninth UV1 vaccination with an increase in IgE towards GM-CSF, but not against UV1 peptides. Hypersensitivity is a rare, but labeled event for sargramostim, and thus, hypersensitivity experienced by this patient was more likely a reaction to the adjuvant rather than to the vaccine.

As telomerase is expressed in hematopoietic cells, skin, and bowel, on-target, off-tumor reactivity constitutes a theoretical side effect. We found no evidence for a shift in side effect profile by targeting telomerase during ipilimumab treatment in this trial. No grade 3-4 hematological toxicities were observed, in line with findings from a randomized phase III trial combining hTERT GV1001 vaccination with chemotherapy in pancreatic cancer (21).

Immune responses in this trial appeared more frequently and rapidly than in patients with prostate cancer (14) and NSCLC (15) receiving UV1 monotherapy. Two patients had spontaneous pre-vaccine immune responses to UV1 peptides. These patients were alive at the time of data censoring and achieved PR and SD as BOR, respectively, indicating a possible clinical advantage of pre-immunity, as has been described in previous reports on melanoma (22) and NSCLC (23). Due to the restricted number of patients and high proportion of immune responders in this trial, a correlation between clinical response and immune responses to specific UV1 peptides could not be established.

As shown previously UV1, peptides are highly immunogenic across different HLA allele types in a Caucasian population (14). This is probably due to a sufficient number of epitopes, ensuring broad population coverage and efficacy of the UV1 vaccine by allowing APCs to select the optimal epitopes for presentation in individual patients. This observation together with the fact that hTERT is expressed in nearly all cancers, supports the universal potential of UV1.

CD4 T cells have been recognized as crucial for effective immunotherapy (7), mainly due to their capacity to enhance cytotoxic T cell responses and re-program the tumor microenvironment. They are also responsible for generating a broad reactivity to new hTERT epitopes (intramolecular epitope spreading) following telomerase peptide vaccination (13). Moreover, ipilimumab has been shown to induce expansion of Th1-like CD4 effector T cells in addition to its effect on exhausted CD8 T cells (7), proposing a suitable companion for a long peptide vaccine inducing CD4 T cell responses. This is a distinct mechanism of CTLA-4 blockade-induced immune response compared to anti-PD-1, where the expansion of tumor-infiltrating exhausted CD8 T cells is believed to be the main mechanism of action (7).

The early-onset and frequent immune responses observed in this trial suggest an effect of CTLA-4 inhibition on the fast expansion of vaccine-specific T cells. This interpretation is strongly supported by data from an animal model investigating gp100 vaccine combined with CTLA-4 checkpoint blockade (24). This report compares different vaccine formulations and elucidates the mechanisms behind reduced tumor control when IFA was used, involving trapping, functional impairment and subsequent destruction of effector T cells at antigen depots with few T cells reaching the tumor (24). Moreover, IFA appeared to create a T cell graveyard at the injection site, where the T cells actually managing to escape apoptosis rapidly became exhausted and had a poor memory formation (25). Thus, this mechanism has been suggested as a possible explanation for the lack of synergy between gp100 vaccine and ipilimumab in the landmark study by Hodi et al. were IFA was used as an adjuvant (2, 24). Interestingly, Hailemichael et al. demonstrated that non-persistent vaccine formulations can reverse these negative effects of depot formulations and act synergistically with CTLA-4 and PD-L1 blockade (24). Data from the current trial extends these results to the clinical setting and points the way to further clinical trials involving combinations of the UV1 vaccine and immune checkpoint blockade.

The distribution of TMB is representative and consistent with what has previously described in patients with metastatic melanoma (26). Thus, the favorable outcomes reported here, as compared to ipilimumab monotherapy, cannot alone be ascribed to a selected population with higher mutation counts conferring improved responses to ipilimumab. The post-treatment reduction in TMB seen in responders is in line with patients treated with PD-1 inhibitors (27) and may reflect killing of tumor cells expressing mutations by neoantigen-specific T cells. We therefore investigated baseline biopsies for the presence of acquired mutations in 29 genes known to be involved in antigen processing and presentation providing immune evasion and primary checkpoint inhibitor resistance (28). Tumors from both responders and non-responders had single nucleotide variants in these genes, and we found no clear correlation between the number or types of genes mutated and response to therapy (**Supplementary Figure S5**). Notably, Li et al. recently identified an association between a higher TMB and TERT mutations, conferring an improved prognosis in patients with metastatic melanoma receiving CTLA-4 blockade, and thus, for combined targeting of telomerase and CTLA-4 in these patients (29).

In this trial, we observed an ORR of 33% of patients, favorable to ipilimumab monotherapy (2, 19, 20). Caution must be taken in interpreting these results, as this trial was not designed to assess the potential superiority as compared to ipilimumab monotherapy, and due to limitations in the number of patients included. As illustrated in **Figure 1**, and previously described for ipilimumab, different patterns of response were observed, including slow onset responses and pseudo-progression. The latter may reflect a slow onset clinical response, reflecting the time it takes for a clinical immune response to evolve, the appearance of a tumor immune infiltrate, edema or an actual increase in tumor burden followed by response (30). One patient who achieved a PR had previously received BRAF-targeted therapy, and one patient, who had previously received dacarbazine, obtained SD, indicating clinical benefits also in second-line treatment.

Median PFS is markedly longer in this trial and the proportion of patients alive at five years substantially higher, than reported previously on ipilimumab monotherapy (2, 19, 20, 31, 32). Although not directly comparable, in an ipilimumab monotherapy phase IV trial at our hospital [NCT02068196], with similar inclusion criteria, 5-year OS was 28% (unpublished). We are aware of the small numbers of patients in the current trial; however, baseline data suggests a representative population.

**Figure 2** summarizes subsequent treatment after progression on study drugs. Notably, seven patients received PD-1 inhibitors after progression. PD-1 checkpoint blockade and BRAF- inhibition have shown OS benefits in metastatic melanoma, and thus, subsequent treatment has influenced patient OS in this trial. However, in a phase II trial reporting on the sequential use of nivolumab after ipilimumab in a similar population, 1-year OS was 54% and the median OS was 16.9 months (33). In comparison, in the current trial, 1-year OS was 75% and the median OS was not reached after 61.0 months follow-up. Moreover, no significant difference in OS was observed in patients with *BRAF* mutated metastatic melanoma that were randomized between nivolumab and investigator’s choice chemotherapy and had progressed after ipilimumab and BRAF-targeted treatment (34). These reports suggest that sequential checkpoint blockade alone cannot explain OS benefits in this trial. However, the UV1-induced immune responses may persist long after end of treatment, long-term survival may be the result of a secondary effect of UV1-specific T cells being released from the PD-1/PD-L1 checkpoint by PD-1 inhibitors.

In conclusion, safety was established, and thus, the primary endpoint was met. Clinical benefit and immune responses observed in this trial are compatible with the known mechanisms of action of the two drugs, suggesting that UV1 and ipilimumab combine favorably by both enabling the clinical potential of the vaccine and providing a broader anti-tumor immune response for an improved effect of ipilimumab. This provides a rationale for combining UV1 with ipilimumab and nivolumab, a current first-line treatment of advanced melanoma. A phase I multi-center trial investigating UV1 in combination with pembrolizumab [NCT03538314] is now fully recruited, and an international randomized trial [NCT04382664] investigating UV1 and ipilimumab combined with nivolumab, versus ipilimumab combined with nivolumab is ongoing.

## Data Availability Statement

The datasets presented in this article are available via the European Genome-Phenome Archive (https://ega-archive.org/) EGA accession number EGAS00001005253. Requests to access the datasets should be directed to espen.ellingsen@ultimovacs.com.

## Ethics Statement

The studies involving human participants were reviewed and approved by Regional Ethical Committee South East. The patients/participants provided their written informed consent to participate in this study.

## Author Contributions

Conception or design of the work: EMI, SA, TG, GG. Data collection: EA, PB, MN, TG. Data analysis and interpretation: EA, EMI, EE, WR, K-MH, DV, SN, EH, TG, GG. Drafting the article: EA, EMI, EE, WR, TG, GG. All authors contributed to the article and approved the submitted version.

## Funding

This work was supported by Ultimovacs ASA, the Norwegian Ministry of Health and Care Services, and the Norwegian Cancer Society [grant number 2220815]. Ultimovacs was involved in the study design of the trial.

## Conflict of Interest

EMI and GG are inventors of a UV1 vaccine patent. EI, WR, GG and SA are shareholders in Ultimovacs ASA. EE, WR, SA and GG are employees of Ultimovacs ASA. MN has received personal honoraria from BMS for lectures.

The authors declare that this study received funding from Ultimovacs ASA. The funder had the following involvement with the study: study design of the trial.

## References

[1] Wei SC Duffy CR Allison JP. Fundamental Mechanisms of Immune Checkpoint Blockade Therapy Vol. 8. Cancer Discov (2018) 8(9):1069–86. 10.1158/2159-8290.CD-18-0367 30115704

[2] Hodi FS O’Day SJ McDermott DF Weber RW Sosman JA Haanen JB. Improved survival with ipilimumab in patients with metastatic melanoma. New Engl J Med (2010) 363(8):711–23. 10.1056/NEJMoa1003466 PMC3549297 20525992

[3] Hellmann MD Ciuleanu TE Pluzanski A Lee JS Otterson GA Audigier-Valette C. Nivolumab plus Ipilimumab in Lung Cancer with a High Tumor Mutational Burden. New Engl J Med (2018) 378(22):2093–104. 10.1056/NEJMoa1801946 PMC7193684 29658845

[4] Snyder A Makarov V Merghoub T Yuan J Zaretsky JM Desrichard A. Genetic basis for clinical response to CTLA-4 blockade in melanoma. New Engl J Med (2014) 371(23):2189–99. 10.1056/NEJMoa1406498 PMC4315319 25409260

[5] Van Allen EM Miao D Schilling B Shukla SA Blank C Zimmer L. Genomic correlates of response to CTLA-4 blockade in metastatic melanoma. Sci (N Y NY) (2015) 350(6257):207–11. 10.1126/science.aad0095 PMC5054517 26359337

[6] Hollingsworth RE Jansen K. Turning the corner on therapeutic cancer vaccines. NPJ Vaccines (2019) 4:7. 10.1038/s41541-019-0103-y 30774998 PMC6368616

[7] Wei SC Levine JH Cogdill AP Zhao Y Anang NAS Andrews MC. Distinct Cellular Mechanisms Underlie Anti-CTLA-4 and Anti-PD-1 Checkpoint Blockade. Cell (2017) 170(6):1120–33.e17. 10.1016/j.cell.2017.07.024 28803728 PMC5591072

[8] Hailemichael Y Dai Z Jaffarzad N Ye Y Medina MA Huang XF. Persistent antigen at vaccination sites induces tumor-specific CD8⁺ T cell sequestration, dysfunction and deletion. Nat Med (2013) 19(4):465–72. 10.1038/nm.3105 PMC3618499 23455713

[9] Vasef MA Ross JS Cohen MB. Telomerase activity in human solid tumors. Diagnostic utility and clinical applications. Am J Clin Pathol (1999) 112(1 Suppl 1):S68–75. 10396302

[10] Zanetti M. A second chance for telomerase reverse transcriptase in anticancer immunotherapy. Nat Rev Clin Oncol (2017) 14(2):115–28. 10.1038/nrclinonc.2016.67 27245281

[11] Teixeira L Medioni J Garibal J Adotevi O Doucet L Durey MD. A First-in-Human Phase I Study of INVAC-1, an Optimized Human Telomerase DNA Vaccine in Patients with Advanced Solid Tumors. Clin Cancer Res Off J Am Assoc Cancer Res (2020) 26(3):588–97. 10.1158/1078-0432.CCR-19-1614 31558479

[12] Suso EM Dueland S Rasmussen AM Vetrhus T Aamdal S Kvalheim G. hTERT mRNA dendritic cell vaccination: complete response in a pancreatic cancer patient associated with response against several hTERT epitopes. Cancer Immunol Immunother CII (2011) 60(6):809–18. 10.1007/s00262-011-0991-9 PMC3098983 21365467

[13] Inderberg-Suso EM Trachsel S Lislerud K Rasmussen AM Gaudernack G. Widespread CD4+ T-cell reactivity to novel hTERT epitopes following vaccination of cancer patients with a single hTERT peptide GV1001. Oncoimmunology (2012) 1(5):670–86. 10.4161/onci.20426 PMC3429571 22934259

[14] Lilleby W Gaudernack G Brunsvig PF Vlatkovic L Schulz M Mills K. Phase I/IIa clinical trial of a novel hTERT peptide vaccine in men with metastatic hormone-naive prostate cancer. Cancer Immunol Immunother CII (2017) 66(7):891–901. 10.1007/s00262-017-1994-y 28391357 PMC11028648

[15] Brunsvig PF Guren TK Nyakas M Steinfeldt-Reisse CH Rasch W Kyte JA. Long-Term Outcomes of a Phase I Study With UV1, a Second Generation Telomerase Based Vaccine, in Patients With Advanced Non-Small Cell Lung Cancer. Front Immunol (2020) 11:572172. 10.3389/fimmu.2020.572172 33324397 PMC7726017

[16] van der Burg SH Arens R Ossendorp F van Hall T Melief CJ. Vaccines for established cancer: overcoming the challenges posed by immune evasion. Nat Rev Cancer (2016) 16(4):219–33. 10.1038/nrc.2016.16 26965076

[17] Eisenhauer EA Therasse P Bogaerts J Schwartz LH Sargent D Ford R. New response evaluation criteria in solid tumours: revised RECIST guideline (version 1.1). Eur J Cancer (Oxford Engl 1990) (2009) 45(2):228–47. 10.1016/j.ejca.2008.10.026 19097774

[18] Birkeland E Zhang S Poduval D Geisler J Nakken S Vodak D. Patterns of genomic evolution in advanced melanoma. Nat Commun (2018) 9(1):2665. 10.1038/s41467-018-05063-1 29991680 PMC6039447

[19] Larkin J Chiarion-Sileni V Gonzalez R Grob JJ Cowey CL Lao CD. Combined Nivolumab and Ipilimumab or Monotherapy in Untreated Melanoma. New Engl J Med (2015) 373(1):23–34. 10.1056/NEJMoa1504030 26027431 PMC5698905

[20] Robert C Schachter J Long GV Arance A Grob JJ Mortier L. Pembrolizumab versus Ipilimumab in Advanced Melanoma. New Engl J Med (2015) 372(26):2521–32. 10.1056/NEJMoa1503093 25891173

[21] Middleton G Silcocks P Cox T Valle J Wadsley J Propper D. Gemcitabine and capecitabine with or without telomerase peptide vaccine GV1001 in patients with locally advanced or metastatic pancreatic cancer (TeloVac): an open-label, randomised, phase 3 trial. Lancet Oncol (2014) 15(8):829–40. 10.1016/S1470-2045(14)70236-0 24954781

[22] Weide B Zelba H Derhovanessian E Pflugfelder A Eigentler TK Di Giacomo AM. Functional T cells targeting NY-ESO-1 or Melan-A are predictive for survival of patients with distant melanoma metastasis. J Clin Oncol Off J Am Soc Clin Oncol (2012) 30(15):1835–41. 10.1200/JCO.2011.40.2271 22529253

[23] Laheurte C Dosset M Vernerey D Boullerot L Gaugler B Gravelin E. Distinct prognostic value of circulating anti-telomerase CD4(+) Th1 immunity and exhausted PD-1(+)/TIM-3(+) T cells in lung cancer. Br J Cancer (2019) 121(5):405–16. 10.1038/s41416-019-0531-5 PMC6738094 31358938

[24] Hailemichael Y Woods A Fu T He Q Nielsen MC Hasan F. Cancer vaccine formulation dictates synergy with CTLA-4 and PD-L1 checkpoint blockade therapy. J Clin Invest (2018) 128(4):1338–54. 10.1172/JCI93303 PMC5873868 29480817

[25] Hailemichael Y Overwijk WW. Cancer vaccines: Trafficking of tumor-specific T cells to tumor after therapeutic vaccination. Int J Biochem Cell Biol (2014) 53:46–50. 10.1016/j.biocel.2014.04.019 24796845 PMC4111967

[26] Chalmers ZR Connelly CF Fabrizio D Gay L Ali SM Ennis R. Analysis of 100,000 human cancer genomes reveals the landscape of tumor mutational burden. Genome Med (2017) 9(1):34. 10.1186/s13073-017-0424-2 28420421 PMC5395719

[27] Riaz N Havel JJ Makarov V Desrichard A Urba WJ Sims JS. Tumor and Microenvironment Evolution during Immunotherapy with Nivolumab. Cell (2017) 171(4):934–49.e16. 10.1016/j.cell.2017.09.028 29033130 PMC5685550

[28] Kelly A Trowsdale J. Genetics of antigen processing and presentation. Immunogenetics (2019) 71(3):161–70. 10.1007/s00251-018-1082-2 PMC6394470 30215098

[29] Li H Li J Zhang C Zhang C Wang H. TERT mutations correlate with higher TMB value and unique tumor microenvironment and may be a potential biomarker for anti-CTLA4 treatment. Cancer Med (2020) 9(19):7151–60. 10.1002/cam4.3376 PMC7541140 32810393

[30] Wolchok JD Hoos A O’Day S Weber JS Hamid O Lebbe C. Guidelines for the evaluation of immune therapy activity in solid tumors: immune-related response criteria. Clin Cancer Res Off J Am Assoc Cancer Res (2009) 15(23):7412–20. 10.1158/1078-0432.CCR-09-1624 19934295

[31] Robert C Ribas A Schachter J Arance A Grob JJ Mortier L. Pembrolizumab versus ipilimumab in advanced melanoma (KEYNOTE-006): post-hoc 5-year results from an open-label, multicentre, randomised, controlled, phase 3 study. Lancet Oncol (2019) 20(9):1239–51. 10.1016/S1470-2045(19)30388-2 31345627

[32] Larkin J Chiarion-Sileni V Gonzalez R Grob JJ Rutkowski P Lao CD. Five-Year Survival with Combined Nivolumab and Ipilimumab in Advanced Melanoma. New Engl J Med (2019) 381(16):1535–46. 10.1056/NEJMoa1910836 31562797

[33] Weber JS Gibney G Sullivan RJ Sosman JA Slingluff CL Jr. Lawrence DP. Sequential administration of nivolumab and ipilimumab with a planned switch in patients with advanced melanoma (CheckMate 064): an open-label, randomised, phase 2 trial. Lancet Oncol (2016) 17(7):943–55. 10.1159/000450974 PMC5474305 27269740

[34] Larkin J Minor D D’Angelo S Neyns B Smylie M Miller WH Jr.. Overall Survival in Patients With Advanced Melanoma Who Received Nivolumab Versus Investigator’s Choice Chemotherapy in CheckMate 037: A Randomized, Controlled, Open-Label Phase III Trial. J Clin Oncol Off J Am Soc Clin Oncol (2018) 36(4):383–90. 10.1016/S1470-2045(15)70076-8 PMC6804912 28671856

