# Supplementary material for: Combining a Universal Telomerase Based Cancer Vaccine With Ipilimumab in Patients With Metastatic Melanoma - Five-Year Follow Up of a Phase I/IIa Trial
Source: Front Immunol. 2021 May 11;12:663865. doi: 10.3389/fimmu.2021.663865 (PMC8147687; doi:10.3389/fimmu.2021.663865)
Supplement: Supplementary file 1 [file DataSheet_1.pdf]

Supplementary data: Sequence analysis

Reads of each sample were mapped (lane-wise) with BWA-mem[1]to human reference genome (build b37 with an added decoy contig, obtained from GATK resource bundle[2]).Sample-wise read sorting and duplicate marking was performed on the initial alignments with Picard tools[3]. GATK tools[4] were subsequently used for two-step local realignment around insertions/deletions (indels), with matching samples (i.e., a tumor and its corresponding normal) being processed together. Each sample's pair-end read information was then checked for inconsistencies with Picard and base-quality recalibration was performed by GATK. Somatic variant calling on the matched sample-pairs was done with MuTect[5] (SNV detection), Strelka[6] (SNV and small indel detection), Manta[7] (large-scale structural variant, medium-sized indel and large insertion detection) and FACETS[8] (CNV analysis). FastQC[9] was used for quality control of analysis input data. GATK tools were used for computing coverage statistics on the recalibrated alignment files. Picard tools were utilized to assess the levels of guanine oxidation in each processed sample.

Resource/reference data

Data	Source	Below referred to as
Human reference genome, build b37 with an added decoy contig	GATK resource bundle 2.8	<implicit use in all analysis steps requiring genome reference>
A reference collection of known indels: 1000 Genomes Phase I indel calls	GATK resource bundle 2.8	*1000G_phase1.indels.b37.vcf*
A reference collection of known indels: Mills and 1000 Genomes indel calls	GATK resource bundle 2.8	*Mills_and_1000G_gold_standard.indels.b37.vcf*
dbSNP variant collection (release 138)	GATK resource bundle 2.8	*dbsnp_138.b37.vcf*
COSMIC variant collection (release 64)	The Cosmic project	*cosmic_v64.b37.vcf*

	website[10]	
List of capture-target regions: Agilent SureSelect Human All Exon V6+COSMIC (S07604715)	Obtained from Agilent Technologies, Inc.[11]	*agilent_S07604715.intervals*
Agilent SureSelect Human All Exon V6+COSMIC (S07604715) capture target regions extended by 250 bp in both directions	Derived locally from the corresponding un-extended region data	*agilent_S07604715_extended.intervals*

Details for the individual analysis steps:

1) <input: lane-wise fastq.gz files generated from sequencer output during demultiplexing>

Mapping of each sample's input reads with BWA mem (version 0.7.8-r455). Performed lane-wise.

The following optional parameters were used:

- -t 20
- -R <sample-specific read group information>
- -M

2) <input: lane-wise SAM output of step 1)>

SAM file coordinate sorting and SAM-to-BAM format conversion. Performed lane-wise for each sample with Picard's 'SortSam' tool (Picard tools version 1.84).

The following optional parameters were used:

- SORT_ORDER=coordinate
- VALIDATION_STRINGENCY=LENIENT
- CREATE_INDEX=true

3) <input: lane-wise BAM output of step 2)>

Merging of lane-wise alignments and marking of duplicate reads, performed individually for each sample with Picard's 'MarkDuplicates' tool (Picard tools version 1.84).

The following optional parameters were used:

- VALIDATION_STRINGENCY=LENIENT
- CREATE_INDEX=true

4) <input: a corresponding pair of BAM files generated in step 3)>

2-step local realignment around indels. Performed for each matched sample pair with GATK's 'RealignerTargetCreator' and 'IndelRealigner' tools (version 2.3-9-ge5ebf34).

The following optional parameters were used with the 'RealignerTargetCreator' tool:

- -known *1000G_phase1.indels.b37.vcf*
- -known *Mills_and_1000G_gold_standard.indels.b37.vcf*
- -L *agilent_S07604715_extended.intervals*
- -nt 20

The following optional parameters were used with the 'IndelRealigner' tool:

- -known *1000G_phase1.indels.b37.vcf*
- -known *Mills_and_1000G_gold_standard.indels.b37.vcf*
- -L *agilent_S07604715_extended.intervals*
- -targetIntervals <output of RealignerTargetCreator's run for the same sample pair>
- -rf NotPrimaryAlignment
- -nWayOut <a text file mapping input- to output- file names>

5) <input: sample-wise BAM output of step 4)>

Application of Picard's 'FixMateInformation' tool on each sample's data (Picard tools version 1.84).

The following optional parameters were used:

- SORT_ORDER=coordinate
- VALIDATION_STRINGENCY=LENIENT
- CREATE_INDEX=true

6) <input: sample-wise BAM output of step 5)>

2-step base-quality recalibration. Performed for each sample with GATK's 'BaseRecalibrator' and 'PrintReads' tools (version 2.3-9-ge5ebf34).

The following optional parameters were used with the ‘BaseRecalibrator’ tool:

- -cov ContextCovariate
- -cov CycleCovariate
- -knownSites *dbsnp_138.b37.vcf*
- -knownSites *1000G_phase1.indels.b37.vcf*
- -knownSites *Mills_and_1000G_gold_standard.indels.b37.vcf*
- -plots <pre-recalibration covariate-plots file for given sample>
- -nct 10

The following optional parameters were used with the ‘PrintReads’ tool:

- -BQSR <output of BaseRecalibrator’s run for the same sample>
- -nct 10

7) <input: a corresponding pair of BAM files generated in step 6)>

Somatic SNV calling, performed on each matched sample pair with MuTect (version 1.1.5).

The following optional parameters were used:

- --cosmic *cosmic_v64.b37.vcf*
- --dbsnp *dbsnp_138.b37.vcf*
- -L *agilent_S07604715_extended.intervals*
- --enable_extended_output

8) <input: a corresponding pair of BAM files generated in step 6)>

Somatic SNV and short indel calling, performed on each matched sample pair with Strelka (version 1.0.13).

Strelka's template configuration file “strelka_config_bwa_default.ini” was used with the following changes:

- value of parameter “isSkipDepthFilters” was set to 1
- value of parameter “isWriteRealignedBam” was set to 1
- value of parameter “indelMaxRefRepeat” was set to 13

The make command which starts Strelka analysis was run with option “-j 20”.

9) <input: a corresponding pair of BAM files generated in step 6)>

Somatic copy number variant identification was performed for each matched sample pair with FACETS (version 0.4.0). The analysis consisted of three main steps:

9.a) compiling genomic loci that would subsequently be interrogated with respect to allelic composition and coverage; this was done by merging variant sites detected in the control sample by MuTect (running MuTect as described in step 7), but with the normal and tumor samples reversed) with a list of SNP sites recommended/provided by the authors of FACETS;

9.b) generating pileup data at the compiled genomic loci with FACETS' own 'snp-pileup' script; default settings were used, utilizing Samtools version 0.1.18[12];

9.c) running the FACETS R code (under R version 3.3.0[13]) with the random number generator seed set to *1234*.

10) < input: a corresponding pair of BAM files generated in step 3)>

Detection of large-scale structural variants, medium-sized indels and large insertions was performed with Manta (Manta workflow version 1.0.0). The configuration script was run the "--exome" option.

The following optional parameters were used with the 'runWorkflow.py' script:

- -m local
- -j 20

11) <input: a corresponding pair of BAM files generated in step 6)>

Coverage statistics were computed for each sample by GATK's 'DepthOfCoverage' tool (version 2.3-9-ge5ebf34).

The following optional parameters were used:

- -omitBaseOutput
- -L *agilent_S07604715.intervals*
- -ct X

The "-ct X" option was used multiple times, with X ranging from 5 to 150 (all multiples of 5 within that range were used).

12) <input: lane-wise fastq.gz files generated from sequencer output during demultiplexing>

Quality control of input fastq.gz files was performed with program FastQC (version 0.10.1). Optional parameter “--noextract” was used.

13) < input: a corresponding pair of BAM files generated in step 6>

To assess the levels of guanine oxidation in each processed sample, Picard’s ‘CollectOxoGMetrics’ tool (Picard tools version 1.84) was run. The investigations were limited to regions present on the "*agilent_S07604715.intervals"* list.

References

1. Li H, Durbin R. Fast and accurate short read alignment with Burrows-Wheeler transform. Bioinformatics 2009; 25: 1754-1760.
2. GATK resource bundle, version 2.8, human reference b37. ftp://gsapubftp-anonymous@ftp.broadinstitute.org/bundle/b37/.
3. Picard tools version 1.84. http://broadinstitute.github.io/picard.
4. McKenna A, Hanna M, Banks E et al. The Genome Analysis Toolkit: a MapReduce framework for analyzing next-generation DNA sequencing data. Genome Res 2010; 20: 1297-1303.
5. Cibulskis K, Lawrence MS, Carter SL et al. Sensitive detection of somatic point mutations in impure and heterogeneous cancer samples. Nat Biotechnol 2013; 31: 213-219.
6. Saunders CT, Wong WS, Swamy S et al. Strelka: accurate somatic small-variant calling from sequenced tumor-normal sample pairs. Bioinformatics 2012; 28: 1811-1817.
7. Chen X, Schulz-Trieglaff O, Shaw R et al. Manta: rapid detection of structural variants and indels for germline and cancer sequencing applications. Bioinformatics 2016; 32: 1220-1222.
8. Shen R, Seshan VE. FACETS: allele-specific copy number and clonal heterogeneity analysis tool for high-throughput DNA sequencing. Nucleic Acids Res 2016; 44: e131.
9. Fast QC version 0.10.1. http://www.bioinformatics.babraham.ac.uk/projects/fastqc/.
10. COSMIC. http://cancer.sanger.ac.uk/cancergenome/projects/cosmic/.
11. Agilent Technologies Inc. http://www.agilent.com.
12. Samtools version 0.1.18. http://samtools.sourceforge.net/.
13. R. http://www.r-project.org.
