# Supplementary material for: Combining a Universal Telomerase Based Cancer Vaccine With Ipilimumab in Patients With Metastatic Melanoma - Five-Year Follow Up of a Phase I/IIa Trial
Source: Front Immunol. 2021 May 11;12:663865. doi: 10.3389/fimmu.2021.663865 (PMC8147687; doi:10.3389/fimmu.2021.663865)
Supplement: Supplementary file 2 [file Image_1.pdf]

Supplementary figure 1

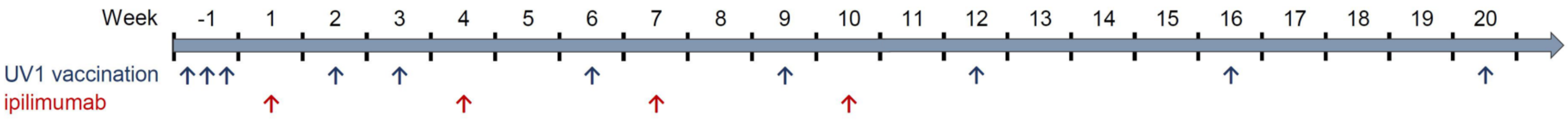

UV1 vaccination and ipilimumab treatment schedule. UV1 and GM-CSF are administered up to 10 times and ipilimumab as labeled
