# Supplementary material for: Combining a Universal Telomerase Based Cancer Vaccine With Ipilimumab in Patients With Metastatic Melanoma - Five-Year Follow Up of a Phase I/IIa Trial
Source: Front Immunol. 2021 May 11;12:663865. doi: 10.3389/fimmu.2021.663865 (PMC8147687; doi:10.3389/fimmu.2021.663865)
Supplement: Supplementary file 3 [file Image_2.pdf]

Supplementary figure S2

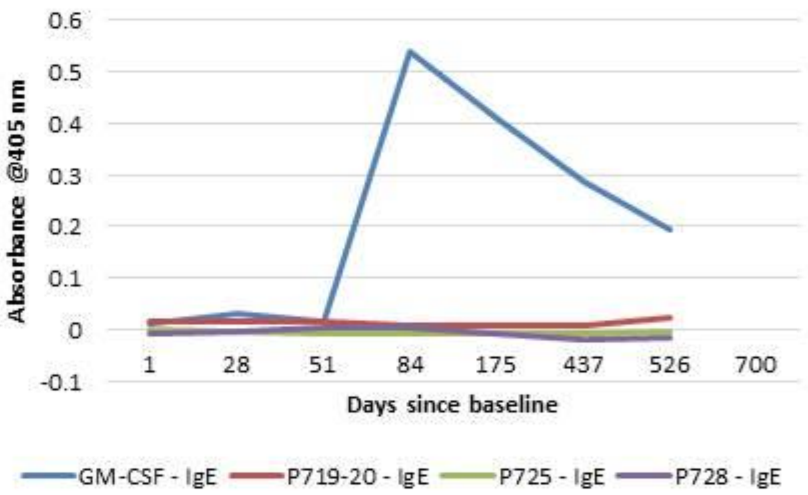

IgE against GM-CSF and UV peptides in one patient. Repeated measurements of IgE against UV1 peptides and GM-CSF in peripheral blood were conducted retrospectively in a patient experiencing a hypersensitivity reaction after the ninth vaccine. An increase in IgE was seen towards GM-CSF, but not towards UV1 peptides. All measurements were done using an Enzyme-linked immunosorbent assays (ELISA)-based in-house method. In brief, MAXISORP 96 well plates (Costar; Cat# 3590) were coated with Leukine (Genzyme; Cat# 011-015) at 5 µg/ml in carbonate/bicarbonate (Sigma; Cat# C3041) buffer (18 h, 4°C), and blocked with 2% human serum albumin (HSA) in washing buffer (PBS, pH 7.4, containing 0.1% (v/v) Tween 20 - Calbiochem) for 1 h at room temperature. Plasma samples (100 µl) were loaded in triplicates and the plates were incubated for 1 h at room temperature. 100 µl goat anti-human IgE-HRP (diluted 1:500 in blocking buffer diluted 1:10 in washing buffer – Sigma-Aldrich) was added and the plates were incubated for 1 h at room temperature. HRP was visualized by the addition of 100 µl ABTS and hydrogen peroxide (9:1 ratio, HRP substrate kit – Bio-Rad). The absorbance at 405 nm was measured using ELISA plate reader. Streptavidin coated plates (Pierce; Cat# 15500) were incubated with biotinylated UV1 peptides (synthesized at Mimotopes) diluted to 200 ng/ml in 0.2% HSA (Octapharma) in PBS-Tween (Calbiochem; Cat# 524653-1EA), and incubated overnight at 4°C. Mouse monoclonal B3102E8 anti human IgE Fc (HRP) (abcam; Cat# ab99806) diluted 1:1000 in 0.2% HSA/PBS-Tween were used as secondary mAb. Peroxidase Substrate kit (BIO-RAD; Cat# 172-1064) were used for detection. Plates were washed three to five times with washing buffer after each step. Due to the absence of standard curve, the results are presented as the absorbance measured at 405 nm normalized to the signal at baseline for each patient.
