# Supplementary material for: Combining a Universal Telomerase Based Cancer Vaccine With Ipilimumab in Patients With Metastatic Melanoma - Five-Year Follow Up of a Phase I/IIa Trial
Source: Front Immunol. 2021 May 11;12:663865. doi: 10.3389/fimmu.2021.663865 (PMC8147687; doi:10.3389/fimmu.2021.663865)
Supplement: Supplementary file 4 [file Image_3.pdf]

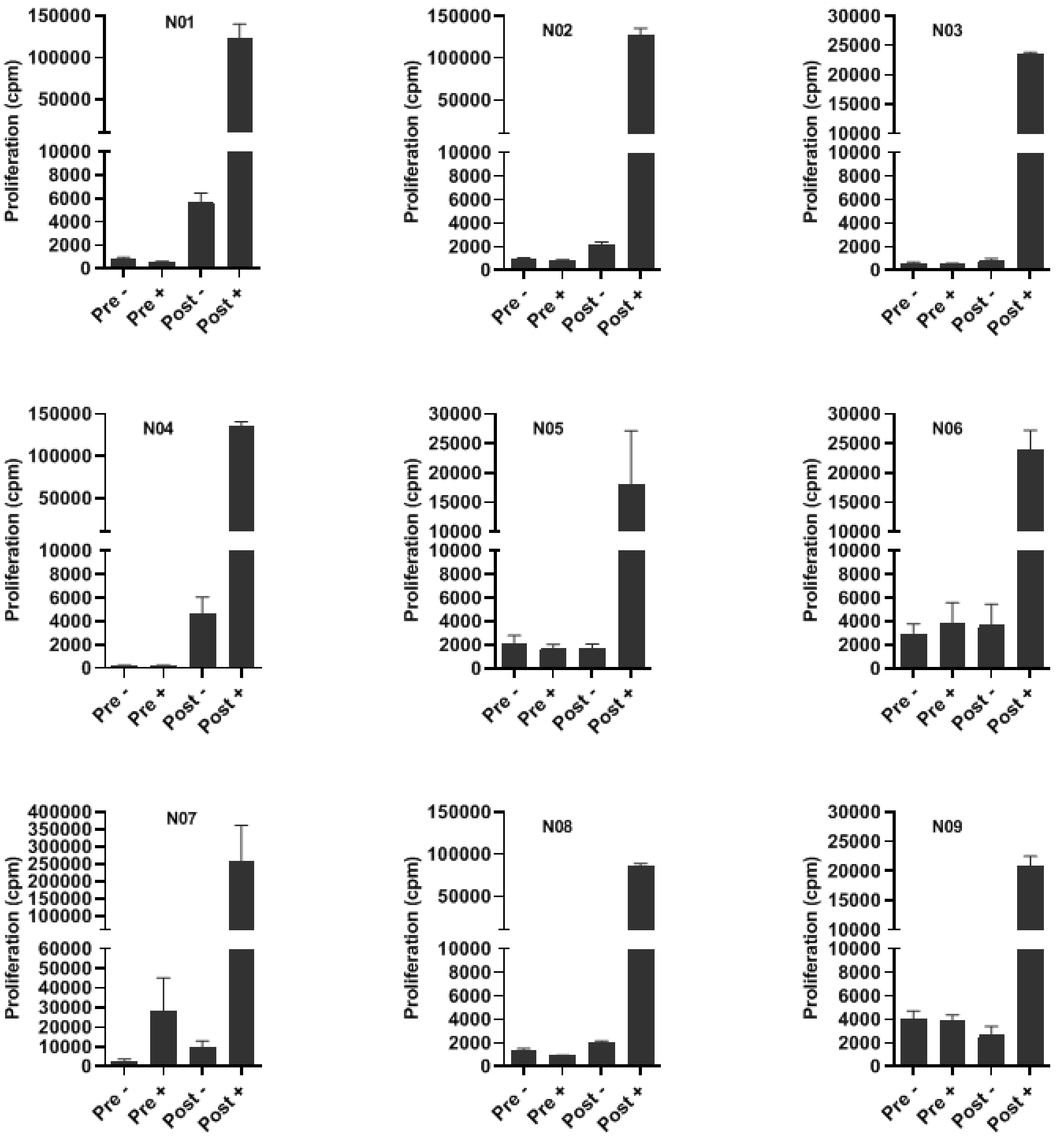

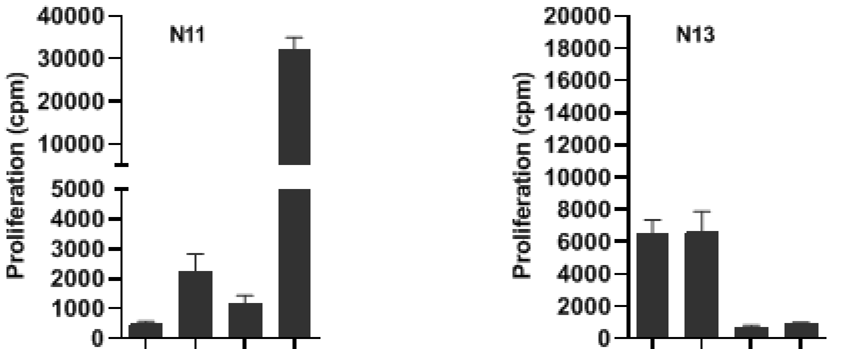

Supplementary Figure S3. Pre- and post--vaccination UV1-specific T-cell responses. T-cell proliferation against UV1 peptides in pre- and post-vaccination blood samples. The graph shows the pre-vaccination and the strongest post-vaccination T-cell responses detected for each patient. Proliferation was measured in response to non-loaded (-) and peptide-loaded (+) PBMC by ^{3}H-thymidine incorporation.
