# Supplementary material for: Combining a Universal Telomerase Based Cancer Vaccine With Ipilimumab in Patients With Metastatic Melanoma - Five-Year Follow Up of a Phase I/IIa Trial
Source: Front Immunol. 2021 May 11;12:663865. doi: 10.3389/fimmu.2021.663865 (PMC8147687; doi:10.3389/fimmu.2021.663865)
Supplement: Supplementary file 5 [file Image_4.pdf]

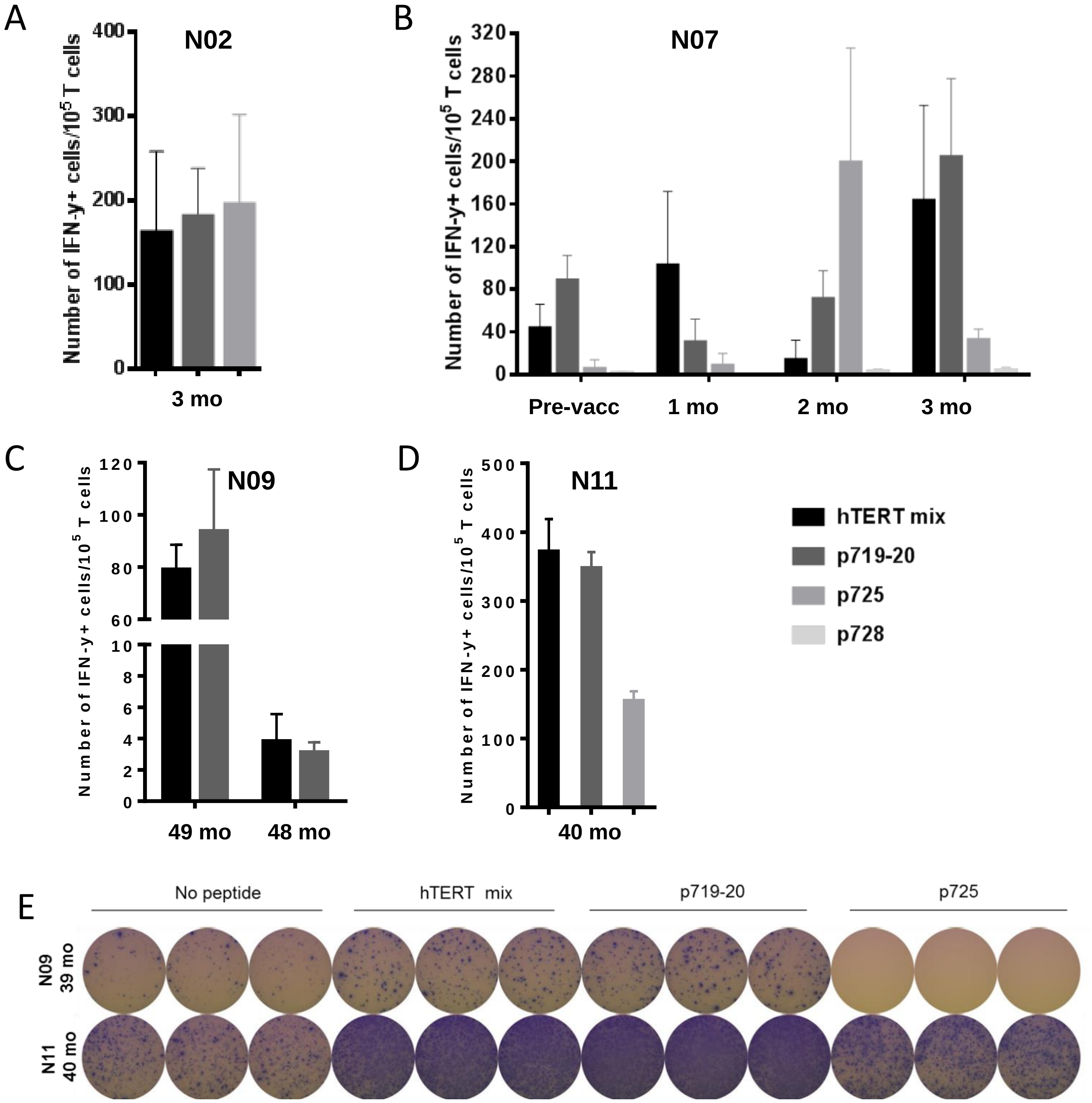

Supplementary Figure S4. UV1-specific IFN-γ secretion by T cells.
IFN-γ secretion was measured on the same UV1-stimulated T cells used for proliferation if sufficient cells numbers at sampling time points allowed for testing in ELISPOT assays. Two representative examples are shown of patient N02 at 3 months post vaccination (A), patient N07 at indicated time points (B), patient N09 at indicated time points (C), and patient N11 at 40 months post-vaccination (D). Responder T cells were seeded in triplicates at 0.1 × 10^6 T cells/well and stimulated with irradiated, autologous PBMCs at an E:T ratio of 2:1. UV1 peptides were added at 10 µM for each peptide. Specific spots were calculated by subtracting the mean number of spots + (2 × SD) of the medium only (no peptide) control from the mean number of spots of experimental wells. E) A representative example of ELISPOT images from patients N09 (low background) and N11 (high background).
