# Supplementary material for: Combining a Universal Telomerase Based Cancer Vaccine With Ipilimumab in Patients With Metastatic Melanoma - Five-Year Follow Up of a Phase I/IIa Trial
Source: Front Immunol. 2021 May 11;12:663865. doi: 10.3389/fimmu.2021.663865 (PMC8147687; doi:10.3389/fimmu.2021.663865)
Supplement: Supplementary file 6 [file Image_5.pdf]

Supplementary figure 5

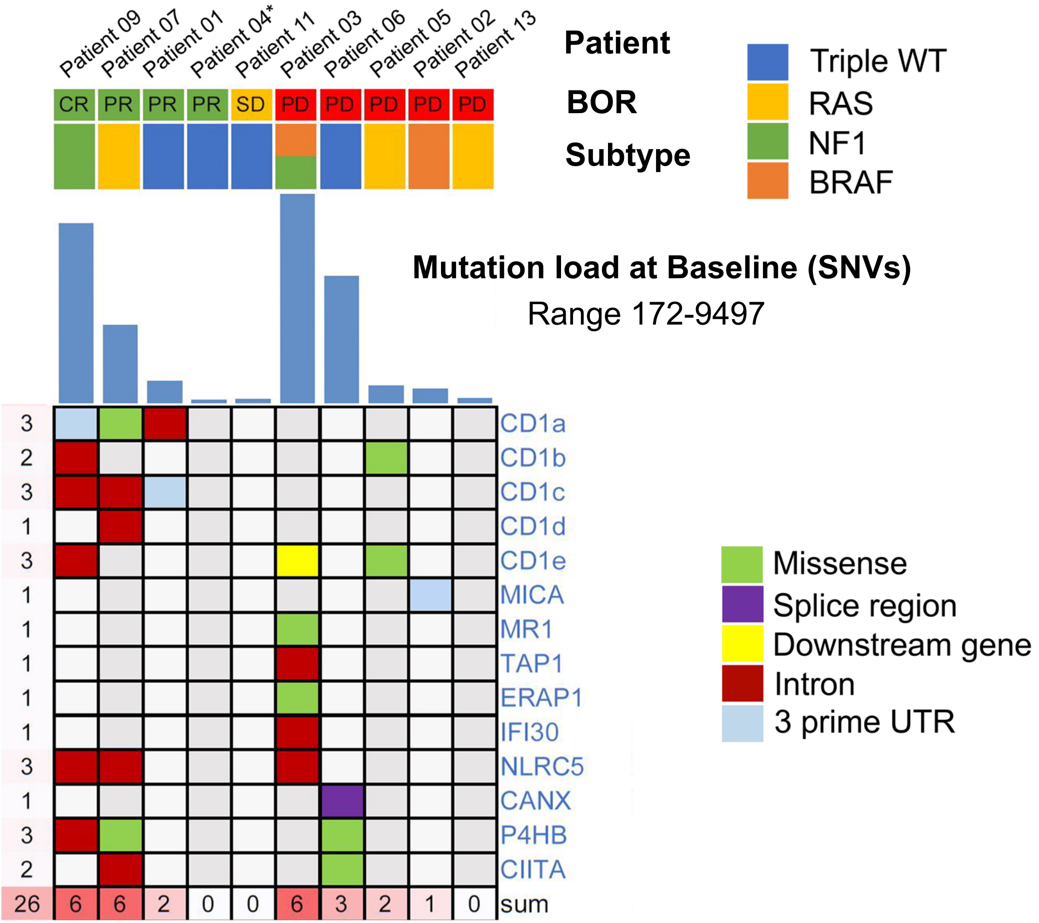

Genomic features of evaluable patients at baseline

Baseline genomic characteristics, sorted by best overall response (BOR) and mutation load (number of single nucleotide variants). The figure depicts variants detected in 29 genes known to be involved in antigen processing and presentation (not including HLA alleles) (non-mutated genes are not shown) (Kelly and Trowsdale, Immunogenetics, 2019).

*Patient 4 only had post-treatment biopsy available.
