# Supplementary material for: Combining a Universal Telomerase Based Cancer Vaccine With Ipilimumab in Patients With Metastatic Melanoma - Five-Year Follow Up of a Phase I/IIa Trial
Source: Front Immunol. 2021 May 11;12:663865. doi: 10.3389/fimmu.2021.663865 (PMC8147687; doi:10.3389/fimmu.2021.663865)
Supplement: Supplementary file 7 [file Table_1.pdf]

Supplementary Table 1. Treatment overview (*N*=12)

Patient	Total number of vaccines	Total number of ipilimumab infusions
N01	9[a]	4
N02	9[b]	4
N03	9[b]	4
N04	7[c]	3[a]
N05	6[b]	3[b]
N06	6[b]	2[b]
N07	5[c]	4
N08	3[c]	3[a]
N09	3[c]	4
N11	3[c]	3[a]
N13	3[c]	3[b]
N14	3[c]	1[d]

Cause of treatment cessation: [a]adverse event, [b]disease progression, [c]regulatory requirements, [d]physician's decision
