# Supplementary material for: Combining a Universal Telomerase Based Cancer Vaccine With Ipilimumab in Patients With Metastatic Melanoma - Five-Year Follow Up of a Phase I/IIa Trial
Source: Front Immunol. 2021 May 11;12:663865. doi: 10.3389/fimmu.2021.663865 (PMC8147687; doi:10.3389/fimmu.2021.663865)
Supplement: Supplementary file 8 [file Table_2.pdf]

Supplementary table 2. Patient HLA typing in relation to UV1 peptides immune responses

HLA-A class I				HLA-A class II						Immune response			
HLA-A		HLA-B		HLA-DRB1		HLA-DQB1		HLA-DPB1		p719-20	p728	p725	UV1 mix
*01:01	*03:01	*07:02	*40:01	*04:04	*15:01	*03:02	*06:02	*03:01P	*04:01P	+	-	-	+
*23:01	*24:02	*35:01	*44:03	*01:03	*07:01	*02:02	*05:01	*02:01	-	+	-	+	+
*03:01	-	*07:02	*15:01	*04:01	*07:01	*02:02	*03:02	*01:01	*04:01	+	-	-	+
*03:01	*25:01	*08:01	*18:01	*03:01	*15:01	*02:01	*06:02	*04:01	-	+	+	+	+
*02:01	*03:01	*44:02	*51:01	*08:03	*15:01	*03:01	*06:02	*02:01	*04:01	+	-	-	+
*01:01	*02:01	*40:01	*57:01	*07:01	*13:02	*03:03	*06:04	*04:02	-	+	-	-	+
*02:01	*03:01	*07:02	*35:01	*07:01	*08:01	*03:03	*04:02	*04:01	-	+	+	+	+
*02:01	*24:02	*15:01	*44:02	*01:01	*13:01	*05:01	*06:03	*03:01P	*04:01P	+	+	+	+
*02:01	*03:01	*07:02	-	*15:01	-	*06:02	-	*04:01	-	+	-	-	+
*02:01	*30:01	*13:02	*44:02	*07:01	*11:01	*02:02	*03:01	*02:01	*17:01	+	-	-	+

Patient HLA typing in immune responders (*N*=10). HLA typing was conducted using Tier 1 Typing by PCR-sequence specific oligonucleotides (PCR-SSOP) to resolve major allele groups to 4 digits, with some degeneracy. Immune responses were observed across HLA subtypes.
